# Multimodal Sensing for Depression Risk Detection: Integrating Audio, Video, and Text Data

**DOI:** 10.3390/s24123714

**Published:** 2024-06-07

**Authors:** Zhenwei Zhang, Shengming Zhang, Dong Ni, Zhaoguo Wei, Kongjun Yang, Shan Jin, Gan Huang, Zhen Liang, Li Zhang, Linling Li, Huijun Ding, Zhiguo Zhang, Jianhong Wang

**Affiliations:** 1 School of Biomedical Engineering, Health Science Center, Shenzhen University, Shenzhen 518060, China; 2100241036@email.szu.edu.cn (Z.Z.); nidong@szu.edu.cn (D.N.); huanggan@szu.edu.cn (G.H.); janezliang@szu.edu.cn (Z.L.); lzhang@szu.edu.cn (L.Z.); lilinling@szu.edu.cn (L.L.); hjding@szu.edu.cn (H.D.); 2 Guangdong Provincial Key Laboratory of Biomedical Measurements and Ultrasound Imaging, Shenzhen 518060, China; 3 Affiliated Mental Health Center, Southern University of Science and Technology, Shenzhen 518055, China; pedestrianm@163.com; 4 Shenzhen Kangning Hospital, Shenzhen 518020, China; rowzag@163.com (Z.W.); yangkongjun0755@163.com (K.Y.); 18249990356@163.com (S.J.); 5 Shenzhen Mental Health Center, Shenzhen 518020, China; 6 School of Computer Science and Technology, Harbin Institute of Technology, Shenzhen 518055, China; 7 Peng Cheng Laboratory, Shenzhen 518055, China

**Keywords:** depression risk detection, emotion elicitation paradigm, multimodal data

## Abstract

Depression is a major psychological disorder with a growing impact worldwide. Traditional methods for detecting the risk of depression, predominantly reliant on psychiatric evaluations and self-assessment questionnaires, are often criticized for their inefficiency and lack of objectivity. Advancements in deep learning have paved the way for innovations in depression risk detection methods that fuse multimodal data. This paper introduces a novel framework, the Audio, Video, and Text Fusion-Three Branch Network (AVTF-TBN), designed to amalgamate auditory, visual, and textual cues for a comprehensive analysis of depression risk. Our approach encompasses three dedicated branches—Audio Branch, Video Branch, and Text Branch—each responsible for extracting salient features from the corresponding modality. These features are subsequently fused through a multimodal fusion (MMF) module, yielding a robust feature vector that feeds into a predictive modeling layer. To further our research, we devised an emotion elicitation paradigm based on two distinct tasks—reading and interviewing—implemented to gather a rich, sensor-based depression risk detection dataset. The sensory equipment, such as cameras, captures subtle facial expressions and vocal characteristics essential for our analysis. The research thoroughly investigates the data generated by varying emotional stimuli and evaluates the contribution of different tasks to emotion evocation. During the experiment, the AVTF-TBN model has the best performance when the data from the two tasks are simultaneously used for detection, where the F1 Score is 0.78, Precision is 0.76, and Recall is 0.81. Our experimental results confirm the validity of the paradigm and demonstrate the efficacy of the AVTF-TBN model in detecting depression risk, showcasing the crucial role of sensor-based data in mental health detection.

## 1. Introduction

Depression is a common mental disorder. Unlike ordinary emotional changes, depression can have profound effects on various aspects of life [1]. Relevant surveys show that approximately 280 million individuals worldwide are currently suffering from depression, and more than 700 thousand people commit suicide annually due to depression [2]. Depression has had a serious impact on human mental health, but its detection has always been a difficult task. Currently, psychiatrists rely mainly on their experience and the self-assessment depression scales to identify subjects at risk for depression. Commonly used self-assessment scales include the Beck Depression Inventory (BDI) and the Patient Health Questionnaire (PHQ). In reality, when individuals fill out the self-assessment scales, they may conceal their real situation. In addition, when the doctor-patient ratio is out of balance, the work pressure of psychologists is great, and the efficiency of detection and the reliability of results cannot be guaranteed.

Facial expressions, pronunciation, intonation, and audio content contain characteristics associated with depression risk. Subjects at risk for depression have fewer facial expressions and mouth movements [3], like to lower their heads [3], and habitually avoid eye contact [4]. In terms of audio, prosodic, source, resonance peak, and spectral features have been used in depression risk detection research [5]. In terms of audio content, the audio content of the subjects at risk for depression in clinical interviews is effective information for detection [6], such as syntax [7] and semantics [8]. If effective features can be extracted from these three types of information, it is expected to achieve high-precision detection. Neural network models built using deep learning technology have outstanding advantages in feature extraction and label prediction. It can automatically learn features from the data and improve its generalization ability through continuous training to perform tasks such as classification or regression.

At present, research on depression risk detection based on audio, video, and text modes has made progress, but there are still several issues that deserve further exploration. First, the robustness and discrimination of the extracted unimodal features need to be improved. Second, high-efficiency fusion methods for multimodal features need to be explored. Some researchers only use simple methods to extract unimodal features and fuse multimodal features [9]. Third, the size of public depression risk detection datasets is generally small, and the original videos are not available, which hinders the ability to extract effective features directly from raw data. The data collection paradigms of public datasets are also relatively simple. For example, the Distress Analysis Interview Corpus-Wizard-of-Oz (DAIC-WOZ) dataset [10] is collected through interviews, but the interview questions are not standardized. This may result in different levels of emotional stimulation among different interviewees. The Audio/Visual Emotion Challenges 2013 (AVEC2013) dataset [11] has a paradigm that includes reading and interviewing tasks. However, the interview questions are too few and not deep enough to elicit emotions.

To address the above issues, this paper conducted experiments and analyses of multimodal data for depression risk detection. We designed an emotion elicitation paradigm consisting of reading tasks and interview tasks, collected facial videos and audio data, and established a multimodal (audio, video, and text) depression risk detection dataset. On the other hand, we propose an AVTF-TBN model that uses three branches to extract features from each modality. An MMF module uses attention and residual mechanisms to fuse multimodal features, which focuses on the importance of each modality while minimizing feature loss. We compare the emotional elicitation effects of different tasks and questions, and the results provide insights into the design of paradigms.

The main contributions of this paper are summarized as follows:

(1) An experimental paradigm for data collection is designed. The paradigm stimulates emotions through reading and interviewing tasks. This paper adopts the experimental paradigm that includes both reading and interviewing tasks to collect data and performs preprocessing on the video, audio, and text data of each subject to establish a multimodal database.
(2) We propose an AVTF-TBN model, which extracts audio, video, and text features using a three-branch architecture. An MMF module fuses the three modality features based on attention mechanisms and residual connections.
(3) The AVTF-TBN model is used to conduct depression risk detection on data from different tasks and questions in our dataset, and we compare the ability of different tasks and questions to stimulate emotions.

The rest of this paper is organized as follows: Section 2 reviews the relevant research on depression risk detection. Section 3 introduces the detailed content of the emotion elicitation paradigm and the collection and processing of the dataset. Section 4 details the framework of the AVTF-TBN model. Section 5 introduces and analyzes the detection results of the AVTF-TBN model. Section 6 summarizes this paper and looks forward to future research directions.

## 2. Related Works

### 2.1. Depression Risk Detection Based on Unimodal Information

#### 2.1.1. Depression Risk Detection Based on Video

Related work has shown that human emotional states can be reflected and expressed through the state of the eyes and a set of interrelated expressions [12]. In the course of a day, only 7% of information is transmitted through language, and 55% of information is transmitted through facial expressions [13]. In terms of facial expressions, subjects at risk for depression have different expression changes [14] and eye movements [15] than healthy people.

In recent years, some research has used tools to manually extract features from expression videos or images [16] and traditional machine learning methods to detect depression risk based on extracted features. Ojala et al. [17] proposed a Local Binary Pattern (LBP) image with illumination invariance and rotation invariance to represent facial texture features. Tadalagi et al. [18] used LBP in their research. Local Binary Patterns on Three Orthogonal Planes (LBP-TOP) [19] and Median Robust Local Binary Patterns from Three Orthogonal Planes (MRLBP-TOP) [20] are variants of LBP. Low-Level Descriptors (LLDs), Face Action Units (FAUs), Head Poses (HP), Eye Gazes (EG), and Landmarks are commonly used facial features. Common machine learning methods include Support Vector Regression (SVR) [20], Support Vector Machine (SVM) [12], Random Forest (RF) [21], Decision Tree (DT) [21], and Logistic Regression (LR) [22].

Deep learning algorithms have made detection more accurate and robust [23]. He et al. [24] proposed a Deep Local Global Attention Convolutional Neural Network (DLGA-CNN) method to predict BDI-II scores. Zhu et al. [23] proposed a dual-branch Deep Convolutional Neural Network (DCNN) model to simultaneously focus on both static and dynamic information of facial expressions. Song et al. [25] converted k-frame facial images into multi-channel amplitude spectra and phase spectra, and then a Convolutional Neural Network (CNN) model was built to predict PHQ-8 scores and sample labels. Niu et al. [26] and Xu et al. [27] used models such as ResNet50 as the backbone network to extract features.

#### 2.1.2. Depression Risk Detection Based on Audio

Research shows that the percentage of speech pauses in the total duration of the audio, speech speed, and pitch variations are all related to depression risk [28]. Many audio features, such as prosody, source, peak, and spectral features, are helpful for depression risk detection. Moore et al. [29] used audio prosody, sound quality, frequency spectrum, and glottal features for depression risk detection. However, feature engineering for audio is very cumbersome. Deep learning methods can extract audio features automatically. Therefore, current research commonly uses deep models such as Long Short-Term Memory (LSTM) and Gated Recurrent Unit (GRU) to extract features from raw audio or Mel-Frequency Cepstral Coefficients (MFCC), spectrograms, LLDs, etc.

Chen et al. [30] proposed a general skeleton called SpeechFormer, which is based on the Transformer for processing audio signals. SpeechFormer extracts audio features in four stages according to the structural patterns of audio, namely frame, phoneme, word, and utterance. This approach greatly reduces computational costs. Zhao et al. [31] proposed a method combining multi-head attention mechanisms in the time dimension with LSTM. The LSTM model can capture long-term dependencies through its gating mechanism. Zhao et al. [32] proposed a dual-branch hybrid network to capture global audio features based on LLD, MFCC, and 3D log Mel spectrograms and used SVR to predict BDI-II scores. MFCC are cepstral parameters extracted in the Mel frequency domain, which is close to the nonlinear human hearing system. Mel spectrograms are a method of audio feature extraction that considers both the temporal, frequency, and spectral information of audio, reflecting human auditory perception and highlighting important features in audio signals. In addition, there are also researchers like Sardari et al. [33] that realize the end-to-end extraction of audio features.

#### 2.1.3. Depression Risk Detection Based on Text

Research has shown that language is an expression of how a person feels and thinks. [34]. Subjects at risk for depression tend to use more negative words and first-person singular pronouns when speaking [35].

Traditional machine learning methods such as SVM and Multi-Layer Perceptron (MLP) cannot extract advanced features such as syntax and semantics from text content. Recently, deep models such as LSTM and Bidirectional Encoder Representations from Transformers (BERT) have shown outstanding performance in the field of natural language processing. The LSTM model can effectively capture contextual information from text data through memory units and gating mechanisms. The BERT model not only captures the contextual information of the text well but also understands the connections between different words and the overall semantic information.

Chiong et al. [36] preprocessed two text datasets collected from Twitter and used LR, Linear Kernel SVM (LSVM), MLP, and DT methods to achieve depression risk detection. The BiLSTM model extracts bidirectional semantic information from text data while preserving long-term relationships between words and repeated contextual information [37]. Additionally, Ansari et al. [38] use both machine learning and deep learning methods for depression risk detection.

### 2.2. Depression Risk Detection Based on Multimodal Information

Actually, the process of detecting subjects at risk for depression by psychologists is a process of synthesizing various information to make decisions. From a professional perspective, depression risk detection itself is a process of fusing multimodal information.

Some research has focused on fusing video and audio modalities. Jan et al. [39] proposed a depression severity prediction system based on audio–video features. In the system, the video part extracts three manual features, which are Edge Orientation Histogram (EOH), LBP, and Local Phase Quantization (LPQ), and uses the VGG-Face model to extract deep features from facial images. The audio part extracts MFCC and spectrogram LLD as audio features. Niu et al. [40] proposed a dual-branch framework that fuses audio–video spatiotemporal features to predict BDI-II scores. This framework uses the LSTM, 2D-CNN, and attention mechanisms, emphasizing audio–video information that is more relevant to depression risk and using a Multimodal Attention Feature Fusion (MAFF) module to further extract complementary features of audio–video features. Dai et al. [41] used 1D-CNN to extract audio features and ResNet50 to extract video features.

As researchers learn more about text data, they realize that the text data also contain relevant information about depression risk. Therefore, some researchers have attempted to fuse audio and text modalities. Solieman et al. [42] proposed a text analysis model and an audio quality model that extract text and audio features based on word vectors of text and the COVAREP audio features set. Shen et al. [43] used the Embeddings from Language Models (ELMo) tool to convert text into high-dimensional sentence embeddings, which were then input into the text branch to extract features. For audio, they first calculate Mel spectrograms and then generate audio embeddings of the same length based on them using the NetVLAD module [44]. Then, the GRU model is used to extract audio features based on the audio embeddings.

Although much research has explored the use of fusing two modalities for depression risk detection, the number of modalities is still low. In principle, reasonably fusing effective information from more modalities can achieve better performance. Currently, research on depression risk detection that simultaneously fuses audio, video, and text modalities is rapidly developing. Fang et al. [45] proposed a Multimodal Fusion Model with a Multi-Level Attention Mechanism (MFM-Att) model to predict PHQ-8 scores. The model includes three branches. The Audio Branch uses the LSTM model to extract features from the COVAREP and FORMANT feature sets. The video branch has the same architecture as the Audio Branch and inputs facial information, which includes head pose, gaze direction, facial action units, and facial 3D coordinates. The Text Branch uses a BiLSTM model and a modal-specific attention mechanism to extract text features from sentence-level embeddings. In the end, the MFM-Att model uses an Attention Fusion Network (AttFN) module to fuse the three modality features. Zheng et al. [46] used a knowledge graph to fuse three modality features, flexibly using modal-specific, modal-internal, and cross-modal attention mechanisms to extract multimodal features. Sudhan et al. [47] used a 2D-CNN to extract facial features, a 1D-CNN to extract audio features, and the BERT model to extract text features.

Overall, in the field of depression risk detection, fusing effective information from multiple modalities simultaneously can improve detection performance. In subsequent research, it may be considered to focus on exploring efficient unimodal feature extraction methods and multimodal feature fusion methods to minimize the loss of features and improve detection performance.

## 3. Datasets

### 3.1. Experimental Paradigm

This paper designed an emotion elicitation paradigm to collect a depression risk detection dataset. The content of the paradigm is shown in Table 1. Data collection is conducted in the form of interviews; the interviewer and the interviewee sit opposite each other with a tripod placed between them. There is a smartphone on the tripod; turn on the camera and point it at the face of the interviewee. Sensors in the camera capture subtle facial expressions and audio characteristics. The resolution of the camera is set to 1080 pixels, and the frame rate is set to 30 frames per second. Before starting data collection, the interviewer needs to explain the content and purpose of data collection to the interviewee and sign the informed consent form with the interviewee.

The paradigm includes two emotional stimulation tasks, which are reading and interviewing. In the reading task, the interviewee needs to read “The Little Match Girl” (fragment). The purpose of the reading is to initially stimulate emotions. After reading, the interviewer follows the paradigm and interviews the interviewee. During the interview, the interviewer asked further questions according to the interviewees’ answers to the first question, and each interviewee was asked the last three questions.

Compared to the paradigms of other datasets, our paradigm has many advantages. First, our paradigm initially stimulates emotions through the reading task, which plays a role in emotional preheating. Our paradigm stimulates deep emotional expression through the interview. Second, the questions set in the interview process are comprehensive and in-depth, aiming to effectively guide the interviewee to reveal their mental state and show more characteristics.

### 3.2. Dataset Collection and Analysis

According to the above paradigm, Shenzhen Kangning Hospital collected the audio and video data of 1911 subjects (Male/Female: 767 subjects/1144 subjects, Age (Mean ± Standard deviation): 34.5 ± 10.6 years), and the label of each subject is the PHQ-9 score. The total score range of the PHQ-9 is 0–27 points, and the subjects are usually classified into two categories based on a cutoff score of 4 points. The score segments and corresponding subject categories are shown in Table 2. Table 3 shows the demographic information of the dataset. There were 621 subjects who were at risk for depression and 1290 healthy subjects.

### 3.3. Dataset Preprocessing

We first preprocess the video data. During video recording, the video contains a large area of irrelevant other areas, such as background. If the other areas are not removed, the model may not be able to pertinently extract the features of the facial area. Therefore, when processing video, the focus is on cropping the facial area of each frame in the video of each subject, removing as much background as possible, and retaining the complete facial area.

**Figure 1:**
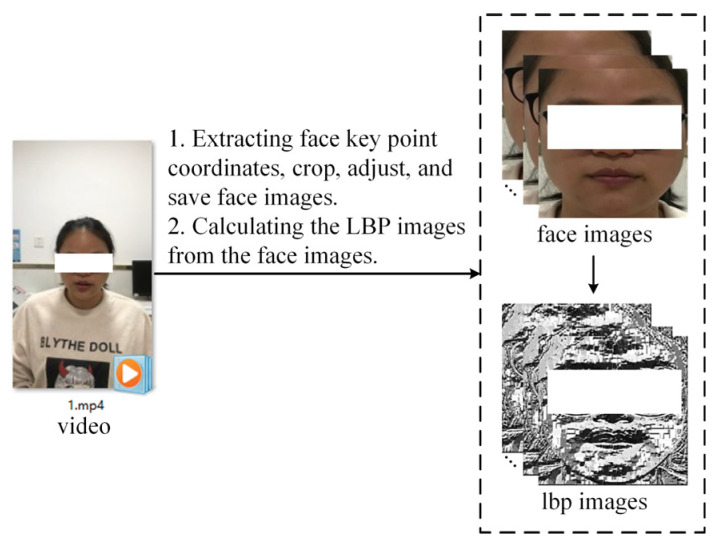
Video processing. The face region is cropped according to the key point coordinates, and then the LBP images corresponding to the face region are calculated.

Specifically, we first used the MediaPipe toolkit to extract key point coordinates on the face. Then, we used OpenCV to crop the face area based on the coordinates, adjust the size of the face area image to 224 × 224, and save it as a JPG file. Figure 1 shows the video processing.

The processing of audio data mainly includes timestamp annotation and the separation of interviewee audio. The original audio contains both the audio of the interviewer and the interviewee. However, only the audio of the interviewee is required for extracting features, so it is necessary to separate it from the original audio. We first manually annotated timestamps of the complete audio files of each subject, including the moments when the subjects began answering different questions and the end moments of answering different questions. The audio of the interviewees was segmented from the original audio based on the timestamps and saved as a WAV file. When extracting MFCC features, we chose 40 filter banks because 40 filter banks are able to cover the frequency range of most speech signals. We utilize a window size of 25 milliseconds and a window overlap of 10 milliseconds to capture the local dynamic changes in the audio signal. We selected a sampling frequency of 16 kHz. In addition, we processed the audio signal by framing it and dividing it into multiple shorter audio frames. Each frame has a length of 25 milliseconds, with a 10-millisecond overlap between frames. Finally, we utilize the Fast Fourier Transform (FFT) to transform each frame from the time domain to the frequency domain.

**Figure 2:**
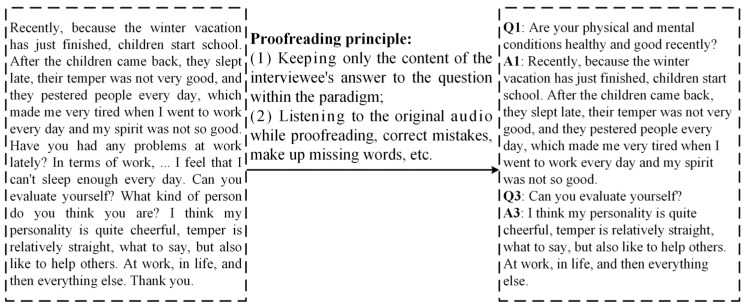
Proofreading transcript text according to the principle.

Text data need to be obtained through audio-to-text conversion and proofreading. First, we used software called Feishu Miaoji (version 7.19) to transcribe the entire original audio of each subject into text. However, there are some missing and incorrect contents in the transcribed text, which need to be further supplemented and corrected. We recruited volunteers to proofread the text. The proofreading principles are mainly two points: one is to retain only the text content of the interviewee’s answers to questions within the paradigm, and the other is to proofread while listening to the original audio. Figure 2 shows a real transcribed text file, along with the proofread text content.

### 3.4. Dataset Usage

Our dataset belongs to the long-tail dataset, which means that as the severity of depression increases, the number of subjects decreases sharply. To construct a balanced dataset, all 120 subjects with PHQ-9 scores greater than 9 (high risk of depression) were included. According to the gender and degree of education of these 120 subjects, 120 healthy subjects were selected to form a dataset containing 240 subjects.

In the use of data, we believe that there are differences in the intensity of emotional expression under different stimulating tasks and questions. Therefore, this paper uses data obtained through the stimulation of different tasks and questions to detect depression risk. Using data in this way can not only fully utilize the dataset but also compare the stimulating abilities of different tasks and questions on emotions. When detection was based on different tasks, the number of subjects was 240. Table 4 shows the answers to the ten questions during the interview process. To ensure that the detection results are representative, we only used data from questions Q1, Q2, Q3, Q4, and Q5 with more than 100 respondents when detecting different problems.

## 4. Methodology

### 4.1. Overview of AVTF-TBN

**Figure 3:**
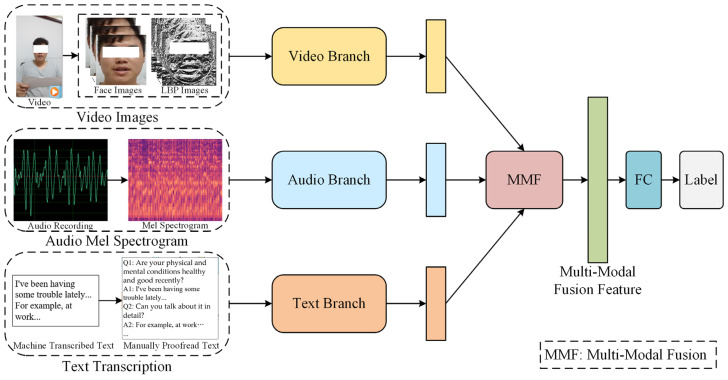
Detailed framework of the AVTF-TBN model. The extracted features of three modalities are fed to the MMF module to be fused. The multimodal fusion feature was used to predict labels.

In this paper, we propose an AVTF-TBN model, the framework of which is shown in Figure 3. The model is a three-branch fusion architecture. First, three separate branches are used to extract the features of video, audio, and text, respectively. Then, an MMF module based on attention and residual mechanisms is used to fuse the features of three modalities to obtain the fusion feature vector. Finally, the labels of the samples are predicted through the full connection layers.

Concretely, the Video Branch extracts global and local facial features from the raw facial images and corresponding LBP maps through the ResNet Swin-Transformer-Dual Branch Network (RST-DBN) module and fuses them to obtain a spatial feature vector of the face also through the module. Then, the branch uses Tanh and Softmax functions to weigh the importance of each frame, uses the BiLSTM model to extract dynamic features of facial expression changes, and uses a Multi-Head Attention (MHA) [48] module to further extract facial features, improving the expressiveness and robustness of the feature vector. The video feature vector can represent the spatiotemporal characteristics of facial expressions. The input of the Audio Branch is the original audio. First, the Mel spectrogram is calculated based on the audio, and then the NetVLAD module is used to obtain a unified-dimensional Mel spectrogram vector. Finally, the GRU model and an MHA module are used to extract audio feature vectors based on it. The input of the Text Branch is raw text data, and the BERT Chinese pre-training model and an MHA module are used to extract text feature vectors. The AVTF-TBN model uses different branches to selectively extract features from three modalities, which helps the model fully extract effective features from each modality. After extracting the feature vectors of the three modalities, the model fuses them through the MMF module, which not only highlights vital information but also preserves the original information in the features of each modality before fusion, thereby improving the performance of the model.

In the experiment in the next chapter, in order to fuse the detection results based on data from two different tasks, we adopt the method of decision fusion for integration. We combined two AVTF-TBN models that used data from two different tasks for depression risk detection to obtain a decision fusion model. When predicting the risk of depression in a certain subject, the model can obtain a prediction probability according to the data from the two different tasks, respectively. After that, the two prediction probabilities are averaged to obtain the final prediction probability of depression risk for the subject.

### 4.2. Video Branch

**Figure 4:**
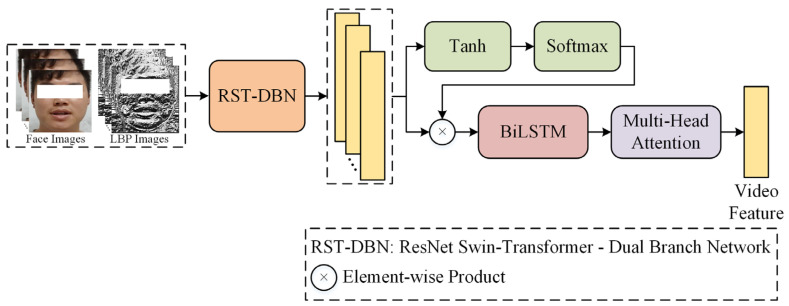
Video Branch framework. The RST-DBN module was used to extract the spatial features of the face. The BiLSTM model was used to extract the temporal features of the face.

**Figure 5:**
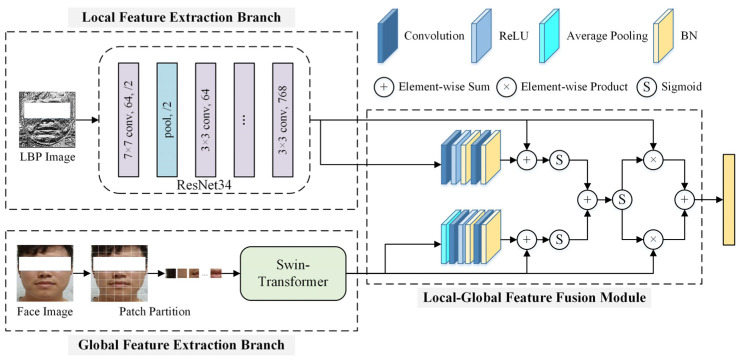
RST-DBN module framework. ResNet34 was used to extract local facial features, and Swin-Transformer focused on global facial information.

The architecture of the Video Branch is shown in Figure 4. Based on the original facial images and corresponding LBP maps, it first extracts static spatial features of facial expressions using the RST-DBN module. The framework of the RST-DBN module is shown in Figure 5, which includes a local feature extraction branch, a global feature extraction branch, and a local–global feature fusion module. The local feature extraction branch uses the ResNet34 model as the backbone network to extract facial texture features from the LBP maps as local features of facial expressions. In the global feature extraction branch, the Swin-Transformer model is used to extract global facial features from the original facial images. The Swin-Transformer model incorporates hierarchical design and sliding window mechanisms, which can pay attention to the local and global features of facial expressions. After extracting local and global features, the local–global feature fusion module is used to fuse the two feature vectors. This fusion module incorporates attention mechanisms and residual connections, fully fusing the two feature vectors while preserving the effective information in the feature vectors before fusion, resulting in more discriminative facial spatial feature vectors.

During depression risk detection, each frame input to the Video Branch contributes differently to detection, and the importance of each frame may vary. Therefore, after the RST-DBN module, the Video Branch calculates weight scores for each frame using Tanh and Softmax functions, then multiplies these weight scores with the spatial feature vectors of corresponding frames to achieve weighting of each frame. Hereafter, considering that facial expressions are dynamic, temporal features in the dynamic process contribute to depression risk detection. Hence, the Video Branch uses the BiLSTM model to further extract dynamic temporal features of expressions, obtaining facial spatiotemporal features that can represent both static and dynamic characteristics of expressions. Finally, the Video Branch uses the MHA module to extract multi-layered facial features based on the spatiotemporal features, resulting in the final video feature vector.

In the Video Branch, the Swin-Transformer model, BiLSTM model, and MHA module are the significant feature extraction modules. The Swin-Transformer model can be divided into four stages, each of which consists of specific network layers. After the image input, it will first pass through a Patch Partition layer, which divides the image into multiple patches. Then, the model performs the first stage of operation. The first stage contains a Linear Embedding layer and several Swin-Transformer blocks, while the other stages each contain a Patch Merging layer and several Swin-Transformer blocks. After passing through four stages, the Swin-Transformer model obtains image feature vectors, which are then used to predict sample labels.

The BiLSTM model consists of a forward LSTM model and a backward LSTM model, which can simultaneously model the forward and backward information of time-series data, making up for the shortcomings of unidirectional LSTM models and providing unique advantages in classification tasks based on long-term time-series data.

After the BiLSTM model, the Video Branch uses the MHA module to extract deep features from the spatiotemporal features. In fact, the MHA module performs multiple scaled dot-product attention operations, and the number of times it is performed is the number of “heads” in the MHA module. The specific working process of the MHA module is as follows:

The MHA module first maps the facial spatiotemporal feature vectors into different vector spaces to obtain Q, K, and V which are vectors of query, key, and value after linear transformation. The output of the single-scaled dot-product attention mechanism in the MHA module can be expressed as Equation (1) [48]. In the equation, the Attention function is used to calculate the output of the self-attention mechanism; the Softmax function calculates the output weights; QK^T^ represents the matrix product; and d represents the dimension of the input vector.

(1) $$Attention\left(Q,K,V\right)=softmax\left(\frac{QK^{T}}{\sqrt{d_{k}}}\right)V,$$

The final output of the MHA module is represented by Equation (2) [48], where ${head}_{i}$ is the output vector of each scaled dot-product attention module in the MHA module, and $W^{O}$ is the output linear transformation matrix. The scaled dot-product attention operation in the MHA module enables the model to capture different patterns and relationships in the input data through the nonlinear transformation of the softmax function. In addition, the MHA module splices the output vectors of different heads together, enabling the model to simultaneously utilize the output information of multiple heads, thereby comprehending the input data more comprehensively.

(2) $$MultiHead\left(Q,K,V\right)=Concat\left({head}_{1},\ldots ,{head}_{h}\right)W^{O},\mathrm{where}{head}_{i}=Attention\left(QW_{i}^{Q},KW_{i}^{K},VW_{i}^{V}\right),$$

### 4.3. Audio Branch

**Figure 6:**
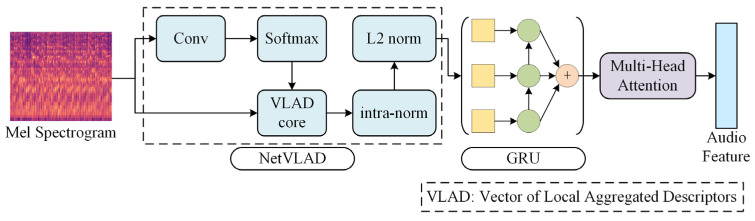
Audio Branch framework.

The framework of the Audio Branch is shown in Figure 6, including the NetVLAD module, GRU model, and MHA module. The NetVLAD module is a convolutional neural network architecture for weakly supervised learning scene recognition that can be embedded into deep models for end-to-end training. Here, it is used to cluster Mel spectrograms of audio for dimensionality reduction and normalizing vectors. The GRU model is used to extract audio timing information. It is a type of RNN that can solve the gradient problem in long-term memory and backpropagation.

The NetVLAD module is an improvement based on the VLAD (Vector of Locally Aggregated Descriptors) module, which is a feature encoding algorithm based on feature descriptors. The module is used to perform dimensionality reduction on Mel spectrograms of different sizes using K-means clustering and normalization to obtain a dimensionally consistent Mel spectrogram vector.

After obtaining the Mel spectrogram vector, the GRU model extracts the temporal features of the audio based on it. The GRU model achieves adaptive selection of information to be forgotten or retained at different time steps through the gating mechanism, ensuring the efficiency of feature extraction. The boxes in the GRU module in Figure 6 represent Mel spectrogram vectors, circles represent GRU units, and the symbol at the end represent the integration of the output of each GRU unit. After the GRU model, the branch also uses an MHA module to extract features in depth, resulting in an audio feature vector.

### 4.4. Text Branch

**Figure 7:**
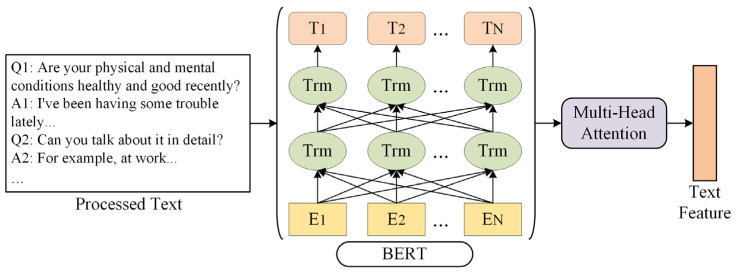
Text Branch framework.

Figure 7 shows the framework of the Text Branch. First, the BERT Chinese pre-training model is used to extract text features. BERT is a transformer-based pre-training language model that captures bidirectional context information for words and sentences. After that, the branch uses an MHA module to deeply extract multi-level text features.

### 4.5. MMF Module

In reality, the process of psychologists conducting a detection with subjects is a process of fusing multimodal information. This paper designs the MMF module based on attention and residual mechanisms to fuse video, audio, and text features.

**Figure 8:**
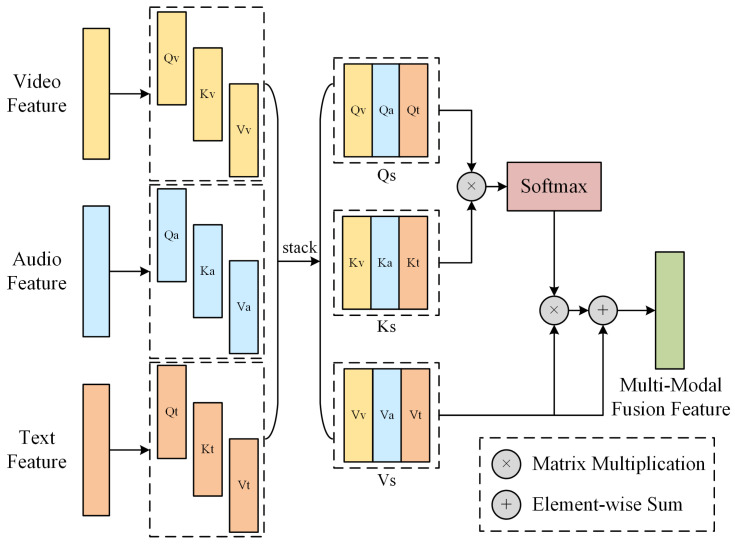
MMF module framework. The module was used to fuse feature vectors of three modalities and to obtain the multimodal fusion feature vector.

The framework of the MMF module is shown in Figure 8. The design of this module was inspired by Zhang et al. [49]. First, the MMF module uses the linear function to map the feature vectors of the three modalities to three different vector spaces, obtaining $Q_{v},K_{v},V_{v},Q_{a},K_{a},V_{a},Q_{t},K_{t},V_{t}$, where v, a, and t represent video, audio, and text, respectively. This step extracts important information from the original feature vectors, facilitating the model to learn more advanced feature representations. Next, the module, respectively, stacks the Q, K, and V, corresponding to the three modalities, to obtain $Q_{s}$, $K_{s}$, $V_{s}$, where s represents stacking, which is equivalent to a simple fusion of different modality features. Then, the module calculates the product of $Q_{s}$ and $K_{s}$, and the product is the similarity matrix between vectors. Subsequently, the similarity matrix is normalized by the softmax function to obtain the weight matrix W, which represents the importance of elements in different positions of the input feature vector. The weight matrix W is used to characterize the importance of the same position elements of feature vectors in different modalities. Finally, the module calculates the product of W and $V_{s}$, and adds the above product to $V_{s}$ to obtain the three-modality fusion vector. The MMF module not only fuses the important information in different modalities according to their importance but also retains the original effective information of each modality before fusion, improving the representation ability of the feature vector and thus improving the performance of the model. The working process of the MMF module is expressed as Equation (3), where σ represents the softmax function, “×” represents the matrix multiplication, and “+” represents the addition of corresponding elements of vectors.

(3) $$MMF=\left(\left(\sigma \left(Q_{s}\times K_{s}\right)\right)\times V_{s}\right)+V_{s}$$

## 5. Experiment Setup and Result Discussion

### 5.1. Experimental Scheme

#### 5.1.1. Implementation Details

In this paper, the AVTF-TBN model was built using the PyTorch framework with version 1.11.0, and the development platformwas an Intel i9-12900K 3.19 GHz processor (Intel, Santa Clara, CA, USA) with a 24.0 GB memory NVIDIA GeForce RTX 3090 graphics card (NVIDIA, Santa Clara, CA, USA). The dataset was stratified into a training set and a test set in a 7:3 ratio. The training set consisted of 168 subjects, and the test set consisted of 72 subjects. The purpose of stratified sampling was to ensure that the proportion of the two types of samples in the training set and test set remained consistent with that of the original dataset, avoiding data imbalance after division. In terms of hyper-parameter settings, the learning rate was set to 1 × 10^−5^, and the AdamW optimizer was used with a weight decay coefficient of 0.01. The model was trained for 300 epochs. The input for the Video Branch was 16 frames, respectively, sampled at equal intervals from all original facial images and LBP maps of each subject, and the model updated its gradients every eight subjects during training.

#### 5.1.2. Evaluation Metrics

This paper calculates three performance metrics, which include Precision, Recall, and F1 Score, based on the confusion matrix to measure the performance of the model. Here, having a depression risk was set as positive, and health was set as negative. TP represents True Positive, which means that the subjects that were truly labeled at risk for depression were predicted to be at risk for depression. TN represents True Negative, which means that the subjects that are truly labeled as healthy are predicted to be healthy. FP represents False Positive, which means that the subjects that were truly labeled as healthy were predicted to be at risk for depression. FN represents False Negative, which means that the subjects that were truly labeled at risk for depression were predicted to be healthy. The calculation equations are shown in Equations (4)–(6), respectively.

(4) $$Precision=\frac{TP}{TP+FP}$$

(5) $$Recall=\frac{TP}{TP+FN}$$

(6) $$F1\;Score=\frac{2\times Precision\times Recall}{Precision+Recall}$$

### 5.2. Experimental Results

#### 5.2.1. Detection Results Based on Different Tasks Data

The AVTF-TBN model first detects based on reading task data, and the results are shown in Table 5. It should be noted that the reading text “The Little Match Girl” (fragment) is uniform, so the text data cannot be used for detection. As shown in the table, when detection is based on unimodal data, the performance of the video modality is generally better than that of the audio modality. This indicates that during the reading process, the facial expressions of subjects provide more features associated with depression risk than audio. When using the MMF module to fuse video and audio modalities, the performance metrics are F1 Score = 0.68, Precision = 0.61, and Recall = 0.78. This reflects that dual-modal fusion can make the model more sensitive to depression risk. Results show that the reading task can preliminarily stimulate emotions.

Then, the model detects based on the interviewing task data, and the results are shown in Table 6. When using unimodal data for detection, the performance of the video modality is generally better, with an F1 Score of 0.65, Precision of 0.66, and Recall of 0.64. In the case of multimodal fusion, the MMF module has the best overall performance for three modality fusions, with an F1 Score of 0.76, Precision of 0.74, and Recall of 0.78. From the results of the dual-modal fusion, the performance is lowest when audio and text modalities are fused. That is, when removing the video modality, the AVTF-TBN model performance decreases the most, indicating that the video modality has the greatest impact on model performance.

We compare the ability of different tasks to provide emotional stimulation according to the results in Table 5 and Table 6. When detection is based on the reading task data, the performance of concatenated video and audio has an F1 Score of 0.66, Precision of 0.65, and Recall of 0.67. This result is slightly lower than the corresponding result when using the interviewing task data, indicating that the fixed questioning during the interview process can stimulate the deep-level emotions of the subjects. At the same time, it must be acknowledged that the reading process before the interview has a preliminary stimulating effect on emotions, and the stimulating effect of the interview process also benefits from the contribution of the reading task. In addition, it can be seen that no matter what task data are used, the performance of the model is better when using the MMF module to fuse feature vectors, proving the effectiveness of the MMF module.

In order to verify the effectiveness of MHA modules in different branches, we conducted ablation experiments based on data from interviewing tasks, and the results are shown in Table 7. It can be seen from the table that when the MHA module is removed, the performance of each branch is slightly decreased, which proves that the MHA module plays a positive role in the feature extraction process of each branch.

Further, we use the decision fusion model for detection based on the data from the two different tasks. The setting of the training parameters of the model is the same as described above. The results are shown in Table 8. Through decision fusion, the detection performance is the best when using the two task data, showing that the F1 Score is 0.78, Precision is 0.76, and Recall is 0.81. This is due to the ability to combine the advantages of the two tasks through fusion, thereby improving performance. This also proves the validity of the paradigm designed in this paper for emotional stimulation.

Table 9 shows the results of the comparison experiment using different task data. Sun et al. [50] proposed the CubeMLP, which is a multimodal feature processing framework based entirely on MLP. The input from three modalities is text content, MFCC, and FAUs, respectively. At first, the framework uses the BERT model to extract text features and LSTM to extract audio and video features. Then, CubeMLP mixes three multimodal features across three axes to obtain the multimodal fusion vectors and flattens them to predict PHQ-8 scores. The Self-Attention Model [51] fuses three modalities of video, audio, and text for emotion recognition. Because there are few open-source codes for multimodal depression risk detection and emotion recognition and depression risk detection belong to the same category of affective computing, we use the emotion recognition model for depression risk detection. By comparison, the AVTF-TBN model has the best performance based on the different task data.

#### 5.2.2. Detection Results Based on Different Questions Data

Table 10 shows the detection results based on three modality data from five questions. The performance based on question Q3 is the most comprehensive, with an F1 Score of 0.67, Precision of 0.54, and Recall of 0.88. The Recall based on question Q1 also reached 0.86. The stem of question Q3 is “Can you evaluate yourself?” We believe that this question can reflect the positive or negative mental state of the subject. The stem of question Q1 is “Are your physical and mental conditions healthy and good recently?” and the interviewer can quickly understand the current mental state of the subject through this question. Additionally, we found that the detection performance based on a single question is lower than that when based on indiscriminating questions (i.e., based on interviewing task data). This indicates that a single question contains limited amounts of information, and fusing information from multiple questions can effectively improve performance.

## 6. Conclusions

In this paper, the AVTF-TBN model is proposed for depression risk detection, and the experimental paradigm is proposed for collecting multimodal depression risk detection datasets. The AVTF-TBN model adopts a three-branch fusion framework to extract features from audio, video, and text data for depression risk detection. The Video Branch extracts local and global features from facial expression images and temporal features from expression changes. The Audio Branch uses the NetVLAD module to convert the Mel spectrogram into vectors and extract audio features using the GRU model. The Text Branch uses the BERT model to extract semantic features from the text content. The MMF module fuses the above features of the three modalities using attention mechanisms and residual connections, obtaining a fused feature vector that is input to the fully connected layers for label prediction. This paper proposes an emotion elicitation paradigm and collects a multimodal dataset. The paradigm includes two kinds of emotion-inducing tasks: reading and interviewing, which can gradually stimulate more expressions and audio information reflecting the emotional state of the subjects. The AVTF-TBN model is detected based on data for different tasks and questions in the dataset. When using the reading task data, the performance indicators for fusing video and audio modalities are the F1 Score of 0.68, Precision of 0.61, and Recall of 0.78. When using the interviewing task data, the performance indicators for fusing video, audio, and text modalities are the F1 Score of 0.76, Precision of 0.74, and Recall of 0.78. We used the decision fusion method to fuse the data of the two tasks for detection, and the performance indicators are the F1 Score of 0.78, Precision of 0.76, and Recall of 0.81. When detection is based on different question data, the comprehensive performance of question Q3 (Can you evaluate yourself?) is the best. The AVTF-TBN model achieves depression risk detection with excellent performance, having the potential for clinical translation and application. In addition, a series of results also prove that the paradigm can effectively stimulate emotions, and various sensors in the camera can be used to capture valid audio and video information. In the future, we will explore the correlation between features of different modalities and reduce model computation, further improving detection performance.

## Figures and Tables

**Table 1:** Emotion elicitation paradigm details.

Emotion Elicitation Paradigm		
Presenter		Contents
		Reading task
Interviewer		Please read the “The Little Match Girl” (fragment).
		Interview task
Interviewer		Are your physical and mental conditions healthy and good recently?
The interviewee answered “No”	Interviewer	Can you talk about it in detail?
	Interviewer	When did this situation start?
	Interviewer	Is the situation worse, better, or completely better now than it was at the beginning?
The interviewee answered, “Yes”	Interviewer	Have you ever had any troubles or pain in the past? If so, can you talk about it?
	Interviewer	How much did it affect your life at that time?
	Interviewer	What were the influences on your work, life, learning, interests, and interpersonal communication at that time?
Interviewer		Can you evaluate yourself?
Interviewer		What kind of person do you think you are in the eyes of the people around you?
Interviewer		What do you think your ideal self is like?

**Table 2:** The corresponding rules for PHQ-9 score segments and subject categories.

PHQ-9 Score Segment	Subject Category
0–4	Health
5–9	Mild depression risk
10–14	Moderate depression risk
15–19	Moderate to severe depression risk
20–27	Severe depression risk

**Table 3:** Four demographic information of the dataset. The values of Age, Height, and Weight are represented as “Mean (standard deviation)”.

	Category	Depression Risk Category	Health Category
Information			
Number of humans (Male/Female)		621 (240/381)	1290 (527/763)
Age		34.6 (10.0)	37.5 (10.7)
Height		164.2 (8.1)	164.0 (8.1)
Weight		61.0 (12.4)	61.4 (13.0)

**Table 4:** Answers cases of different questions within the paradigm.

Question Number	Problem Content	Number of Respondents
Q1	Are your physical and mental conditions healthy and good recently?	240
Q1.1	Can you talk about it in detail?	47
Q1.2	When did this situation start?	51
Q1.3	Is the situation worse, better, or completely better now than it was at the beginning?	50
Q2	Have you ever had any troubles or pain in the past? If so, can you talk about it?	140
Q2.1	How much did it affect your life at that time?	81
Q2.2	What were the influences on your work, life, learning, interests, and interpersonal communication at that time?	46
Q3	Can you evaluate yourself?	205
Q4	What kind of person do you think you are in the eyes of the people around you?	172
Q5	What do you think your ideal self is like?	127

**Table 5:** Detection results of the AVTF-TBN model based on reading task data.

Model ^1^	Data Sources	Modality ^2^	F1 Score	Precision	Recall
	Reading task	Unimodal Data			
AVTF-TBN		V	0.65	0.62	0.69
		A	0.62	0.63	0.61
		Multimodal Data			
AVTF-TBN (Concatenate)		V + A	0.66	**0.65**	0.67
AVTF-TBN (MMF)		V + A	**0.68**	0.61	**0.78**

^1^ “Concatenate” represents the horizontal concatenation of feature vectors of different modalities. “MMF” represents using the MMF module to fuse feature vectors of different modalities. ^2^ KEY-V: video; A: audio; T: text. The best results in each column are in bold font—the same as below.

**Table 6:** Detection results of the AVTF-TBN model based on interviewing task data.

Model	Data Sources	Modality	F1 Score	Precision	Recall
	Interview task	Unimodal Data			
AVTF-TBN		V	0.65	0.66	0.64
		A	0.62	0.63	0.61
		T	0.64	0.64	0.64
		Multimodal Data			
AVTF-TBN (Concatenate)		V + A	0.67	0.57	**0.81**
		V + T	0.67	0.62	0.72
		A + T	0.66	0.68	0.64
		V + A + T	0.74	0.70	0.78
AVTF-TBN (MMF)		V + A + T	**0.76**	**0.74**	0.78

**Table 7:** Performance verification result of the MHA module.

Model	Data Sources	MHA	Modality	F1 Score	Precision	Recall
AVTF-TBN	Interview task	Yes	V	**0.65**	**0.66**	**0.64**
			A	0.62	0.63	0.61
			T	0.64	0.64	**0.64**
		No	V	0.63	0.62	**0.64**
			A	0.61	0.61	0.61
			T	0.62	0.61	**0.64**

**Table 8:** Comparison of detection results based on different task data.

Models	Data Sources	Modality	F1 Score	Precision	Recall
AVTF-TBN (MMF)	Reading task	V + A	0.68	0.61	0.78
	Interview task	V + A + T	0.76	0.74	0.78
	Reading task + Interview task	V + A + T	**0.78**	**0.76**	**0.81**

**Table 9:** Results of the comparison experiment.

Models	Data Sources	Modality	F1 Score	Precision	Recall
CubeMLP [50]	Reading task	V + A	0.56	0.53	0.59
Self-Attention Model [51]			0.57	0.54	0.61
AVTF-TBN(MMF) (Ours)			**0.68**	**0.61**	**0.78**
CubeMLP [50]	Interview task	V + A + T	0.67	0.70	0.64
Self-Attention Model [51]			0.70	0.71	0.69
AVTF-TBN(MMF) (Ours)			**0.76**	**0.74**	**0.78**
CubeMLP [50]	Reading task + Interview task	V + A + T	0.68	0.70	0.66
Self-Attention Model [51]			0.73	0.72	0.74
AVTF-TBN(MMF) (Ours)			**0.78**	**0.76**	**0.81**

**Table 10:** Detection results of the AVTF-TBN model based on different question data.

Model	Data Sources	Modality	F1 Score	Precision	Recall
AVTF-TBN (MMF)	Q1	V + A + T	0.65	0.53	0.86
	Q2		0.61	0.56	0.67
	Q3		**0.67**	0.54	**0.88**
	Q4		0.63	**0.60**	0.67
	Q5		0.65	0.57	0.77

## Data Availability

The author of this paper does not have the authority to publicly disclose the dataset.
